# Health Functions and Related Molecular Mechanisms of Tea Components: An Update Review

**DOI:** 10.3390/ijms20246196

**Published:** 2019-12-08

**Authors:** Guo-Yi Tang, Xiao Meng, Ren-You Gan, Cai-Ning Zhao, Qing Liu, Yi-Bin Feng, Sha Li, Xin-Lin Wei, Atanas G. Atanasov, Harold Corke, Hua-Bin Li

**Affiliations:** 1 Guangdong Provincial Key Laboratory of Food, Nutrition and Health, Department of Nutrition, School of Public Health, Sun Yat-Sen University, Guangzhou 510080, China; tanggy5@mail2.sysu.edu.cn (G.-Y.T.); mengx7@mail2.sysu.edu.cn (X.M.); zhaocn@mail2.sysu.edu.cn (C.-N.Z.); liuq248@mail2.sysu.edu.cn (Q.L.); 2 School of Chinese Medicine, Li Ka Shing Faculty of Medicine, The University of Hong Kong, No. 10 Sassoon Road, Pokfulam, Hong Kong 999077, China; yfeng@hku.hk (Y.-B.F.); lishaha@hku.hk (S.L.); 3 Department of Food Science & Technology, School of Agriculture and Biology, Shanghai Jiao Tong University, Shanghai 200240, China; weixinlin@sjtu.edu.cn (X.-L.W.); hcorke@sjtu.edu.cn (H.C.); 4 Institute of Urban Agriculture, Chinese Academy of Agricultural Sciences, Chengdu 610213, China; 5 The Institute of Genetics and Animal Breeding, Polish Academy of Sciences, Jastrzębiec, 05-552 Magdalenka, Poland; atanas.atanasov@univie.ac.at

**Keywords:** tea, *Camellia sinensis*, phytochemicals, catechins, health benefits, bioavailability, safety

## Abstract

Tea is widely consumed all over the world. Generally, tea is divided into six categories: White, green, yellow, oolong, black, and dark teas, based on the fermentation degree. Tea contains abundant phytochemicals, such as polyphenols, pigments, polysaccharides, alkaloids, free amino acids, and saponins. However, the bioavailability of tea phytochemicals is relatively low. Thus, some novel technologies like nanotechnology have been developed to improve the bioavailability of tea bioactive components and consequently enhance the bioactivity. So far, many studies have demonstrated that tea shows various health functions, such as antioxidant, anti-inflammatory, immuno-regulatory, anticancer, cardiovascular-protective, anti-diabetic, anti-obesity, and hepato-protective effects. Moreover, it is also considered that drinking tea is safe to humans, since reports about the severe adverse effects of tea consumption are rare. In order to provide a better understanding of tea and its health potential, this review summarizes and discusses recent literature on the bioactive components, bioavailability, health functions, and safety issues of tea, with special attention paid to the related molecular mechanisms of tea health functions.

## 1. Introduction

**Figure 1:**
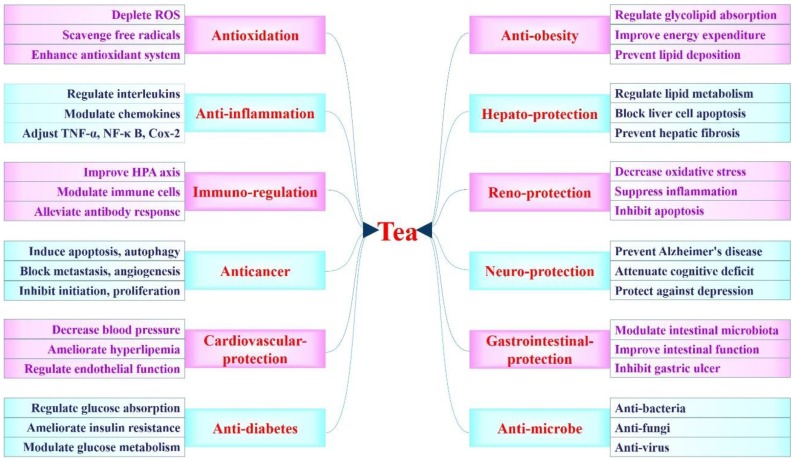
The main health functions of tea.

Tea, a beverage prepared from the leaves of *Camellia sinensis*, originated in ancient China and has become increasingly popular all over the world in recent decades [1]. According to the complex production processes, tea can be classified into six categories, including white, green, yellow, oolong, black, and dark teas. White and green teas are not fermented, yellow tea is just slightly fermented, while oolong, black, and dark teas are more deeply fermented [2,3,4,5,6,7]. Tea contains various bioactive components, such as polyphenols, pigments, polysaccharides, alkaloids, free amino acids, and saponins [8,9,10,11,12,13]. In addition, many studies have indicated that tea and its bioactive components possess multiple health functions (as shown in Figure 1), including antioxidation, anti-inflammation, immuno-regulation, anticancer, cardiovascular-protection, anti-diabetes, anti-obesity, and hepato-protection [14,15,16,17,18,19]. Moreover, several technologies, including recently developed nanotechnology, have been adopted to improve the bioavailability of tea polyphenols [20,21,22,23,24]. Furthermore, the adverse effects of tea were seldom observed [25,26,27,28,29]. Thus, the combination of health functions and safety of tea supports its consumption for people with the potential to prevent and manage certain chronic diseases, such as obesity and cancer.

In order to provide a comprehensive understanding of tea, in this review, its bioactive components, bioavailability, health functions, and safety are summarized and discussed mainly based on in vitro, in vivo, and clinical studies, with highlighting the molecular mechanisms of health functions. Overall, this review can be helpful for the better utilization of tea as beverages and functional foods to prevent and control certain chronic diseases.

## 2. Bioactive Components

Many bioactive components have been identified in tea and its brewing, including polyphenols, pigments, polysaccharides, alkaloids, free amino acids, and saponins, and the amount of these compounds can be quite different in different categories of tea [8,9,10,11].

### 2.1. Polyphenols

White, green and yellow teas contain abundant polyphenols, especially catechins and their derivatives, including catechin, epicatechin (EC), gallocatechin (GC), epigallocatechin (EGC), catechin gallate (CG), epicatechin gallate (ECG), gallocatechin gallate (GCG), and epigallocatechin gallate (EGCG) [12,13,30,31,32]. In addition, other polyphenols like gallic acid, chlorogenic acid, ellagic acid, galloylquinic acid, kaempferol-3-*O*-glucoside (kaempferol-3-G) and various flavonoids are also found in tea [12,13,33,34,35]. Polyphenols have been reported to exhibit various health functions in vitro and in vivo [30,31,32,33,34,35]. Specifically, tea polyphenols are one of the most important natural antioxidants [30]. The antioxidant capacity of tea polyphenols can be influenced by the spatial configuration, and generally positively correlate with the number of hydroxyl groups [32,36].

### 2.2. Pigments

During fermentation, tea catechins are oxidized to theaflavins, thearubigins, and theabrownins, therefore, oolong, black, and dark teas are rich in pigments [4,37,38]. The structures of theaflavins, which have been identified with 4 isomers, including theaflavin, theaflavin-3-gallate, theaflavin-3′-gallate, and theaflavin-3,3′-gallate, are simpler than those of thearubigins and theabrownins that are complex mixtures of polyphenols and their polymers [39,40]. Tea pigments have also been shown as important bioactive components responsible for health functions of tea, like anti-inflammatory, anticancer, and hepato-protective effects, though their antioxidant activity may be lower when compared with tea catechins [10,17,41].

### 2.3. Polysaccharides

Tea polysaccharides (TPS) is another main bioactive component of tea other than polyphenols [42]. The content of polysaccharides in tea could be increased as the maturity of raw tea leaf increased, quite different from the pattern of tea polyphenols [42]. In addition, TPS have diverse chemical characteristics, in terms of the monomer (mainly glucose, galactose, rhamnose, and arabinose, with little xylose and mannose), acidity (neutral or acidic), solubility (water-soluble or not), and conjugation with proteins, polyphenols, metal ions, selenium, strongly influencing the structure–function relationship [42,43,44,45,46,47]. For example, the complex of tea polysaccharides with lower content of polyphenols exert higher antioxidant activity than those with higher content of polyphenols, and conjugation with selenium could remarkably increase the antioxidant activity of tea polysaccharides [42,46]. Polysaccharides may contribute to the antioxidant, immuno-regulatory, anticancer, anti-diabetic, and anti-obesity effects of tea brewing and its extracts [44,47,48,49,50].

### 2.4. Alkaloids

Tea is one of the most important sources of alkaloids, generally as purine alkaloids (e.g., caffeine, theobromine, and theophylline), which can be transformed into flavo-alkaloids [8,51]. A possible pathway has been proposed to involve deamination, decarboxylation, and spontaneously cyclization of L-theanine, and then attachment of the product to EGCG form the flavo-alkaloids [51]. Caffeine is the most abundant alkaloid in all six categories of tea [8]. The antioxidant, anti-diabetic, and anti-obesity effects of tea alkaloids have been described in some studies [30,48,51].

### 2.5. Amino Acids

Tea brewing and its extract also contain a considerable amount of amino acids [52]. Aspartic acid, glutamic acid, arginine, alanine, tyrosine, and theanine have been reported as the major amino acids in tea, and the amino acid profile can be changed during fermentation [8,29,52]. Among them, theanine is a nonproteinic amino acid special to tea [8]. It has been summarized that L-theanine has positive effects on relaxation, cognitive performance, emotional status, sleep quality, cancer, cardiovascular diseases, obesity, and the common cold [8,29].

### 2.6. Saponins

Saponins are another bioactive component in tea brewing and its extract, and usually exhibit antioxidant, immuno-regulatory, anticancer, and cardiovascular-protective effects [11,53,54,55]. Moreover, tea saponins are generally regarded as safe compounds that have anti-fungal and insecticidal properties and are widely used in the field of agriculture and food industry [56,57].

**Figure 2:**
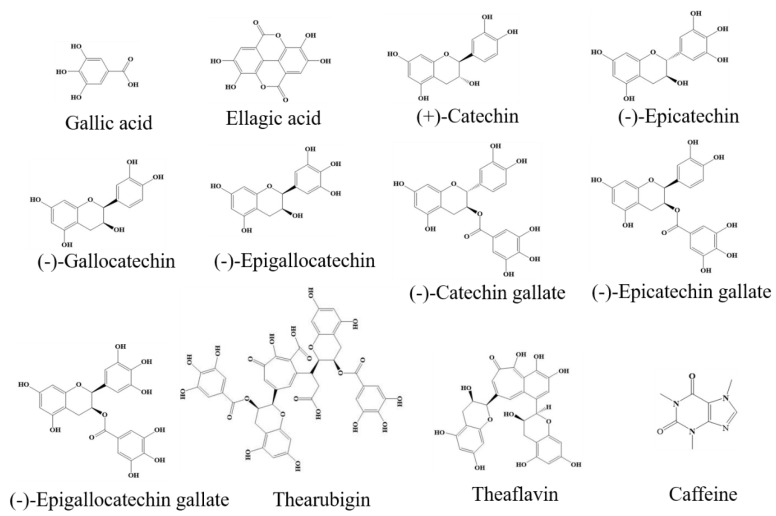
Chemical structures of several bioactive compounds in tea.

Phytochemical contents of 6 representative tea samples from six categories are summarized in Table 1. Eight catechins, caffeine, theaflavine, gallic acid, chlorogenic acid, ellagic acid, and kaempferol-3-G are the main chemical compounds in tea [12,13]. In addition, the chemical structures of main phytochemical compounds in tea are shown in Figure 2.

## 3. Bioavailability

Tea and its bioactive components show various biological activities and health functions, which are strongly correlated with their bioavailability, which are used in this review to designates the quantity or fraction of the ingested dose that is available to organisms, tissues or cells. So far, investigations have mainly focused on the bioavailability of tea polyphenols, such as catechin, EC, ECG, EGC, and EGCG.

### 3.1. Bioavailability of Tea Polyphenols

Many factors, regarding absorption, metabolism, distribution and excretion in the body, can influence the bioavailability of tea polyphenols [23,58,59]. Generally, the bioavailability of tea polyphenols is relatively low, mainly due to the low rate of absorption through gastrointestinal tract [60]. For example, it was reported that < 2% of the EGCG dose given orally was available in the systemic blood in rats [60]. Whereas, the absorption rate of oolong and black tea polyphenols is higher than that of green tea polyphenols [61]. To be more specifically, except for a few phenolic compounds like chlorogenic acid that can be absorbed in the stomach, tea polyphenols are mainly absorbed in the intestine [62,63,64,65]. Thus, proposed strategy to enhance the absorption of tea polyphenols and subsequently improve their bioavailability should target on intestine. Moreover, tea polyphenols are degraded and catabolized before absorption in the intestine, especially in the small intestine, in which gut microbiota plays a crucial role in the metabolism of tea polyphenols [63,64,65]. Hydrolyzed, methylated, sulfated, and glucuronidated actions are deemed as the common process of tea polyphenol metabolism by gut microbiota [64,65,66,67]. Metabolites of tea polyphenols are transported in the circulatory system and distributed in a wide range of organs and tissues, in which they contribute to the health functions of tea. These metabolites are mainly excreted in the urine and feces with apparently high recovery rate and short terminal elimination half-life, which is also responsible for the low bioavailability of tea polyphenols [59,68,69]. Nevertheless, some internal organs can still be exposed to non-marginal doses of tea polyphenols and their metabolites up to 24 h and even 48 h, partly explaining the health functions of tea [64,69].

### 3.2. Strategies to Increase Tea Polyphenol Bioavailability

Tea polyphenols are unstable in oxygen, acidic, and alkaline environment. Overcoming of these may help to improve their bioavailability, so some techniques, like modification technology, capsule technology, and nanotechnology, have been applied to achieve this [20,21,22,23,24]. Modifying tea polyphenols with peracetate acid can protect the free hydroxyl groups surrounding the molecules, improving their stability, and consequently result in increased bioavailability [20]. Delivery systems using protein-, lipid-, and carbohydrate-based carriers and/or capsules can not only reduce the instability of tea components, but also enhance their solubility, ensure favorable slow and sustainable release, and elevate the permeation in the small intestine, resulting in an increased concentration in the plasma and improved bioavailability and biological efficacy [21,22,23,24]. Moreover, fermentation could significantly increase the bioavailability of oolong, black and dark teas compared with unfermented counterpart, which may attribute to metabolism of tea components during fermentation by microbes like bacteria, yeasts, and fungi [70]. Furthermore, some dietary factors, including sucrose, ascorbic acid, piperine, quercetin, red onion, and *Dendropanax morbifera*, have been found to improve the digestion, metabolism, absorption, plasma concentration, bioaccessibility, and elimination half-life of tea and its components, all of which lead to the elevated bioavailability [71,72,73,74,75].

### 3.3. Factors That Reduce Tea Polyphenol Bioavailability

There are some factors that may reduce the bioavailability of tea polyphenols, such as ingestion on a non-empty stomach or with dietary proteins. For example, the area under the plasma concentration-time curve in healthy humans consuming EGCG capsules on an empty stomach was 2.7 and 3.9 times higher than that in counterparts consuming EGCG capsules with a light breakfast (*p* = 0.044) or consuming EGCG embedded in the strawberry sorbet (*p* = 0.019), respectively [76]. Similar actions were also observed for the plasma maximum concentration and mean concentration during the dosing interval, indicating the inhibition of tea catechin absorption when it is consumed with breakfast or with strawberry sorbet [76]. These results are consistent with another study based on 30 healthy volunteers, demonstrating that greater bioavailability of tea catechins could be achieved by consuming the Polyphenon E (a decaffeinated and defined green tea catechin mixture) capsules on an empty stomach after overnight fasting [77]. Moreover, it was found that simultaneous ingestion of dietary proteins from milk, caseinate, or soy significantly reduced the bioavailability of galloylated catechins (ECG and EGCG) and total catechins from green tea in humans, though the bioavailability of nongalloylated catechins (EC and EGC) was increased [78]. The difference of galloylated and non-galloylated catechins in bioavailability could be because of some kind of competition between individual catechins, and the complexation of galloylated catechins with proteins could delay liberation/absorption of these catechins, which would promote the absorption of non-galloylated catechins. Furthermore, dietary pretreatment with green tea EGCG (3.2 mg/g diet) for 2 weeks reduced the bioavailability of subsequent oral bolus doses of EGCG in CF-1 mice [79].

In short, tea polyphenols generally have a relatively low bioavailability, which mainly involves the digestion, metabolism, absorption, distribution, and excretion in the body. Many factors can impact the bioavailability of tea and its bioactive components, such as their own physicochemical properties, fermentation techniques, dietary factors, dosing conditions, species diversity, and individual differences. On the other hand, it lacks evidence about the bioavailability of other components in tea brewing and its extract, such as pigments, polysaccharides, saponins, and amino acids in the literature. In the future, more attention should be paid to the bioavailability of other components of tea.

## 4. Health Functions

### 4.1. Antioxidant Activity

In the literature, some studies have reported the antioxidant activity of tea brewing, extract and its components, which may have the potential for management of oxidative stress-induced diseases [80,81,82].

#### 4.1.1. Antioxidant Activity In Vitro

The antioxidant activity of tea brewing, extract and its components has been evaluated by several in vitro biological assay methods using cellular antioxidant activity (CAA), erythrocyte hemolysis, and plasma oxidation assays [80,81,82]. Zeng et al. assessed the CAA of 27 tea cultivars, and the CAA values were 37.7–134.3 μmol quercetin equivalent (QE)/g dry weight (DW) (11.4–40.6 mg QE/g DW) without phosphate buffer washing, and 25.3–75.4 μmol QE/g DW (7.6–22.8 mg QE/g DW) with phosphate buffer washing [80]. The CAA assay is a more biologically relevant method compared to the chemistry assays, since it considers the uptake, metabolism, and distribution of antioxidant components in cells [80]. Moreover, Liu and Huang assessed the antioxidant activity of black tea extract using erythrocyte hemolysis, plasma oxidation, and CAA assays, showing that black tea extract dose-dependently protected erythrocytes from 2, 2′-azobis (2-amidinopropane)-induced oxidative hemolysis and copper-induced plasma oxidation, and the tea pigments, especially thearubigins and theabrownins, mainly contributed to the antioxidant activity of black tea extract [81]. The mechanisms of the cellular antioxidant assay may include restraining the generation of reactive oxygen species (ROS) by inducing the antioxidant enzyme activities, decreasing thiobarbituric acid-reactive substances (TBARS) and peroxyl radicals by avoiding dichlorofluorescein oxidation, increasing 2′, 7′-dichlorofluorescein production, and blocking lipid peroxidation of low-density lipoprotein (LDL) and high-density lipoprotein (HDL) [81].

#### 4.1.2. Antioxidant Activity In Vivo

The antioxidant activity of tea extract and its component has also been investigated in vivo [83,84]. The water extracts of green, black and dark teas were found to improve the tolerance of *Caenorhabditis elegans* to the Cr^6+^-induced oxidative stress [83]. Among these teas, green tea extract showed antioxidant activity probably by regulating the dietary restriction and germline signaling pathways in *C. elegans*, but not the forkhead box O (FOXO) and mitochondrial respiratory chain signals [83]. In addition, green tea extract could improve the oxidative stress status in mice by increasing content of plasmatic SH-groups like reduced glutathione (GSH) and improving antioxidant enzymes in tissues, including NADPH quinone reductase in liver and small intestine, thioredoxin reductase in small intestine and superoxide dismutase (SOD) in liver [84].

#### 4.1.3. Antioxidant Activity in Humans

In humans, tea extract and its component have shown to protect against oxidative stress-related injury [85,86,87]. Administration of green tea extract prevented the oxidative stress-mediated by repeated cycle sprint tests in sprinters [85]. Yabukita and Benifuuki green tea could protect against cutaneous oxidative stress by increasing the radical scavenging activity of the skin [86]. In mildly hypercholesterolemic subjects, green and oolong tea extracts enriched with catechins could significantly improve the content of GSH and the activity of antioxidant enzymes, including SOD, catalase (CAT), glutathione peroxidase (GPX), and glutathione reductase (GR) [87].

Collectively, tea extracts show good antioxidant activity mainly due to its diverse antioxidant components, such as polyphenols, polysaccharides, and pigments, which can scavenge free radicals, deplete ROS, increase antioxidant contents, and enhance antioxidant enzyme activities.

### 4.2. Anti-Inflammatory Activity

The considerable anti-inflammatory activity of tea and its bioactive components has been demonstrated with insights into the multiple mechanisms of action, indicating the potential in treating and managing inflammatory related diseases [88].

#### 4.2.1. Anti-Inflammatory Activity In Vitro

Several research groups have investigated the in vitro anti-inflammatory activity of tea and related mechanisms. Cyboran et al. illustrated that green tea extract exerted a strong anti-inflammatory activity on red blood cells with no evident toxic effect [89]. Ben Lagha and Grenier demonstrated that black tea theaflavins attenuated the virulence of *Porphyromonas gingivalis*, regulated the tight junction integrity of the gingival keratinocytes, and exhibited an anti-inflammatory activity, showing the potential of preventing and treating periodontal inflammatory disease, which had multiple mechanisms, including the downregulation of inflammatory factors by *P. gingivalis*-stimulated macrophages, such as interleukin (IL)-1, IL-6, tumor necrosis factor α (TNF-α), chemokine (C-X-C) ligand 8, matrix metalloprotease (MMP)-3, MMP-8, and MMP-9, attenuation of the *P. gingivalis*-induced activation of the nuclear factor κB (NF-κB) signaling pathway, and inhibition of the gelatin degradation mediated by MMP-9 [90]. In addition, green tea supplements offered an anti-inflammatory effect in primary human rheumatoid arthritis synovial fibroblasts, in which catechins (EC, EGC, and EGCG) from green tea were found to have different impacts [91]. EGCG and EGC restrained IL-6, IL-8, and MMP-2 production and selectively suppressed COX-2 expression, while EC did not show any inhibitory activity on these factors. The three catechins could block the key signaling protein in the IL-1β-signaling pathway, namely TAK-1, the transforming growth factor (TGF)-β-activated mitogen-activated protein kinase (MAPK). But only EGCG was able to occupy the major part of the active site of TAK-1. Moreover, EGCG could also inhibit the protein expression of p38 and NF-κB, whereas EC and EGC did not. These results suggest that EGCG and EGC can be the main contributors to the anti-inflammatory effect of green tea, and EGCG is the most powerful catechin to inhibit the downstream signaling of inflammation.

#### 4.2.2. Anti-Inflammatory Activity In Vivo

The protective activity of tea against inflammation has been further assessed in vivo [17,92,93]. Ramadan et al. investigated the anti-inflammatory activity of green tea extracts (rich in catechins) and black tea extracts (rich in theaflavins and thearubigins) in adjuvant-induced arthritic rats with two doses (0.5 and 1.0 g/kg BW), and found that green tea extracts at 1.0 g/kg remarkably alleviated arthritis in rats, accompanied with ameliorating synovial joint inflammation, elevating erythrocyte sedimentation rate, and restoring weight/cellularity of lymphoid organs [17]. These effects might be mediated by the downregulation of systematic pro-inflammatory cytokines and synovial tissue chemokine receptor-5. Additionally, Liu et al. reported that 4-week pretreatment with tea polyphenols (300 mg/kg BW) significantly alleviated the inflammation mediated by acute exhaustive exercise in rats, and the serum levels of pro-inflammatory factors including TNF-α, IL-1β, and IL-6 were significantly reduced in rats fed with tea polyphenols, accompanied with a shift of the serum IL-10/TNF-α ratio to a predominantly anti-inflammatory milieu and a suppression of IL-1β mRNA expression in the liver [92]. Moreover, green and black teas (70 mg/kg BW) and their related components, such as EGCG (10 mg/kg BW), theaflavins (9 mg/kg BW), and caffeine (18 mg/kg BW), effectively protected against murine sepsis, which could lie in modulating neutrophil influx and preventing neutrophil accumulation in lungs, decreasing systematic TNF-α and IL-6, suppressing tissue inducible nitric oxide synthase (iNOS) and COX-2, and increasing IL-10 [93].

#### 4.2.3. Anti-Inflammatory Activity in Humans

Recent clinical trials have investigated the anti-inflammatory effect of tea [94,95,96]. A randomized, double-blinded, placebo-controlled clinical trial indicated that the daily consumption of green tea extracts (1000 mg, two capsules/day) for 12 weeks significantly improved the systemic lupus erythematosus (SLE) disease as well as the corresponding vitality and general health [94]. The results from another clinical trial suggested that green tea (tea leaves, 12 g/day) and coffee (approximately 300 mg/day) had similar effects regarding decreasing plasma levels of inflammatory factors, such as IL-6 and NF-κB, in soldiers with sleep deprivation, but green tea had the advantage of maintaining this effect [95]. However, it seemed that the acute ingestion of green tea and carbohydrate (catechins, 22 mg/kg BW; caffeine, 6 mg/kg BW; glucose, 230 mg/kg BW; fructose, 110 mg/kg BW) did not evidently improve inflammatory biomarkers during sprint cycling in athletes in comparison to carbohydrates (glucose, 230 mg/kg BW; fructose, 110 mg/kg BW) [96].

In short, tea extracts and its bioactive components possess strong anti-inflammatory activity, thus can be a potential agent for arthritis, sepsis, and SLE, with the mechanisms mainly including the regulation of pro-inflammatory and anti-inflammatory factors, like interleukins, chemokines, TNF-α, NF-κB, and COX-2, as well as the related signaling pathways.

### 4.3. Immuno-Regulatory Activity

The immuno-regulatory activity of tea brewing, extract and its bioactive components has been widely evaluated, with the mechanisms of action discussed below.

#### 4.3.1. Immuno-Regulative Activity In Vitro

The disequilibrium of different CD4^+^ T-cell subpopulations, including Th1, Th2, Th17, and Treg cells with specific function in immune and inflammatory responses, plays a crucial role in the pathogenesis of autoimmune diseases. EGCG has been reported to inhibit the multiplication and cell cycle progression of naive CD4^+^ T-cells, and to block naive CD4^+^ T-cell differentiation into Th1 and Th17 effector subsets [97]. In another in vitro study, the results suggested that anthocyanins-enriched tea also exhibited immuno-stimulatory activity [98].

#### 4.3.2. Immuno-Regulative Activity In Vivo

Tea and its bioactive components also exhibit systematic and peripheral immuno-regulatory activities in vivo. Wang et al. reported that the immunity of dairy cows was improved after a 6-week administration of tea saponins (0, 20, 30, and 40 g/d) [11]. Sharma et al. declared that EGCG (100 mg/kg BW) from green tea enhanced the systemic immunity in aged male Swiss albino mice by improving the cellular immune response and simultaneously alleviating the antibody response aided by increased adrenal dehydroepiandrosterone [99]. It was observed that EGCG remarkably increased the plasma dehydroepiandrosterone level, the eosinophil and monocyte accounts in blood, the fraction of CD3^+^ CD8^+^ cells in splenocytes, and the CD28 expression on peripheral blood mononuclear cells, while it decreased the secretory IgA and IgE as well as the IgG1/IgG2a ratio. In addition, treatment with 5-(3′, S′-dihydroxyphenyl)-γ-valerolactone (10 mg/kg BW), the major metabolite of EGCG, not only increased the activity of CD4^+^ T-cells but also enhanced the cytotoxic activity of natural killer (NK) cells [100]. Moreover, black tea (10.48 mg solid content/kg BW) showed a protective effect on the peripheral immune responses in rats injected with intracerebroventricular colchicine, regarding higher phagocytic activity of the white blood cells and the splenic polymorphonuclear cells, and higher cytotoxicity and lower leukocyte adhesion inhibition index of the splenic mononuclear cells [101]. Furthermore, Ahmed et al. demonstrated that green tea by-products (contain 9.22% moisture, 20.1% crude protein, 2.91% crude fat, 18.2% crude fiber, 4.88% crude ash, 33.2% nitrogen-free extract, and 11.6% catechins) supplemented at the ratio of 0.5%, 1.0%, or 2.0% in diet positively modulated the proliferation of immune cells in goats in a linear mode [102]. On the other hand, tea can interact with other natural products to regulate the immune response [50,103]. In one study, the mixture of green tea and grape seed extract (100: 200 mg/kg BW) relieved the immune suppression induced by γ-irradiation in male rats, showing radioprotective effect [103]. In another study, the combined administration of green tea Se-TPS and Huo-ji polysaccharides (1:1, 300 mg/kg BW) exerted synergistic effects on improving the immune function in mice [50].

#### 4.3.3. Immuno-Regulative Activity in Humans

In a human study, green tea polyphenol administration (2 × 350 mg/day, for 14 days) has been reported to decrease the level of IgE in patients with allergic rhinitis compared with those in the control group, though not statistically significant [104]. Since evidence is limited, more clinical trials are warrant in this field to further elucidate the immuno-regulative activity of tea extracts and its components.

In summary, tea extracts and its bioactive components, especially catechins, possess immuno-regulatory activity to improve both systematic and peripheral immunity, mainly by modulating immune cell proliferation, differentiation, activation, and alleviating antibody response, as well as regulating the hypothalamus-pituitary-adrenal (HPA) axis. Although tea does not have an evident effect on allergic rhinitis, it has the potential to manage immune-mediated diseases like autoimmune and encephalomyelitis.

### 4.4. Anticancer Effect

#### 4.4.1. Anticancer Effect In Vitro

**Figure 3:**
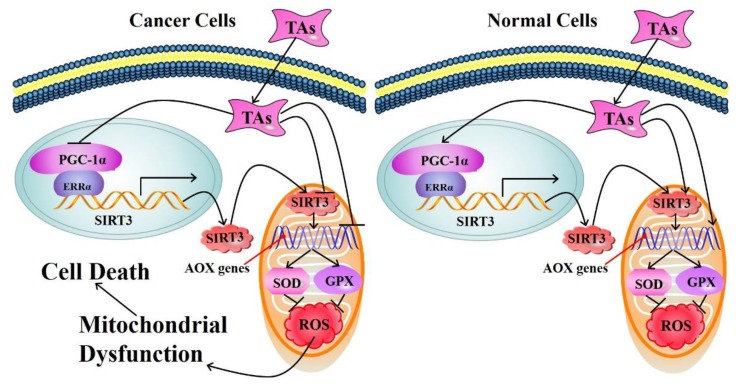
The molecular mechanisms of tea antioxidants (TAs) with contrasting influences on cancer and normal cells. In cancer cells, TAs inhibit the expression and activity of sirtuin 3 (SIRT3), leading to mitochondrial reactive oxygen species (ROS) accumulation, mitochondrial dysfunction, and ultimately cell death. In normal cells, TAs activates SIRT3 and related downstream antioxidant responsive genes (AOX genes, including superoxide dismutase 2 (SOD2) and glutathione peroxidase 1 (GPX1)), preventing cells from oxidative damage. Abbreviations: ERRα, estrogen-related receptor α; PGC-1α, peroxisome proliferator-activated receptor γ coactivator 1α.

The anticancer activity of tea extracts and its components has been widely investigated in vitro. Park et al. reported that green tea rhamnogalacturonan-II-type polysaccharide (GTR-II) could inhibit tumor metastasis [44]. GTR-II enhanced the tumoricidal activity of macrophages and NK cytotoxicity against Yac-1 tumor cells. On the other hand, injection of rabbit anti-asialo GM1 serum could lead to the depletion of NK cells, which in turn eliminated the prohibitory activity of GTR-II on B16BL6 melanoma cells. These results together indicate that the anticancer effect of GTR-II can be mediated by the activated macrophages and NK cells. Krstic et al. demonstrated that green tea extracts possessed anticancer activity on HeLa human cervical adenocarcinoma cells, which depended on the pro-oxidant and anti-proliferative activities of polyphenols [105]. Furthermore, tea components have different effects on cancer cells and normal cells. It was shown that black tea pigments exerted potent inhibitory activity against cisplatin-resistant ovarian cancer cells, while they were less cytotoxic to normal ovarian cells, with the mechanisms involving the induction of G1 cell cycle arrest by down-regulating cyclin-dependent kinase (CDK) 2, CDK4, and cyclin E1, and mediating apoptosis through p53-dependent, ATM/Chk/p53, Akt, and MAPK pathways [10]. In another study, EGCG showed different pro-oxidative effects on normal and oral cancer cells, which was correlated with a different regulation of the sirtuin (SIRT)-3 pathway [106]. ROS in mitochondria was induced by EGCG in SCC-25 and SCC-9 human oral squamous carcinoma cells and MSK-Leuk1 premalignant leukoplakia cells, but not in HGF-1 normal human gingival fibroblast cells. In addition, EGCG inhibited SIRT-3 mRNA and protein expression as well as SIRT-3 activity, reduced the nuclear localization of estrogen-related receptor α, a SIRT-3 transcription regulator in SCC-25 cells, while enhanced SIRT-3 activity in HGF-1 cells. Moreover, EGCG could differentially modulate the mRNA expression of SIRT-3-associated downstream antioxidant-responsive genes, including GPX1 and SOD2, in oral cancer cells and normal cells, with the related molecular mechanisms shown in Figure 3.

#### 4.4.2. Anticancer Effect In Vivo

Tea and its components also exhibited anticancer activity in vivo [3,107,108,109]. Calgarotto et al. found that green tea (100 mg/kg BW) possessed anticancer effect in HL-60 human leukemia xenograft mice, by reducing tumor growth via mediating G1 phase cell cycle arrest, mediating apoptosis via the regulation of caspase-3, Bcl-2 (B-cell lymphoma 2), Bcl-xL (B-cell lymphoma-extra large), Bax (Bcl-2-associated X protein), MCL-1, LC3-I, and LC3-II, and initiating autophagic progression via the activation of autophagy proteins [107]. Torello et al. reported that green tea (250 mg/kg BW) could induce anti-leukemic activity in an acute promyelocytic leukemia model, which was triggered by the production of ROS, activation of caspase-3/8/9, and nuclear translocation of HIF-1α [108]. In addition, green tea polyphenon-60 (250 mg/kg BW) exerted an apoptogenic effect against Ehrlich’s ascites carcinoma cells in Swiss albino mice [109]. Moreover, Kujawska et al. demonstrated that yellow tea extract (10 g/kg feed) protected the liver of rats from N-nitrosodiethylamine-induced hepatocarcinogenesis via its antioxidant effect as revealed by the reversion of SOD, CAT, GPX, paraoxonase 1, and reduced glutathione (GSH), which in turn decreased lipid peroxidation, protein carbonyl formation, and DNA degradation [3].

#### 4.4.3. Anticancer Effect in Humans

Several clinical trials also demonstrated the anticancer effect of tea and its components [110,111,112]. It was reported that green tea consumption (5 × 1 cup/day, four weeks) changed oral bacteria, which might be related to oral carcinogenesis [110]. In another clinical trial involving 70 Algerian prostate cancer patients and 120 age-matched healthy subjects, daily consumption of 5 cups of infusion prepared from 2 g green tea for 6 months significantly decreased oxidative stress and prevented prostate cancer initiation [111]. On the other hand, in a short-term double-blinded placebo-controlled phase II clinical trial with 60 high-grade prostate intraepithelial neoplasia patients, consumption of green tea catechins (600 mg/d) showed no significant difference in prostate cancer incidence between the experimental and control groups after 6 and 12 months, but a non-significant improvement in lower urinary tract symptoms and a better quality of life with very limited adverse effects were observed [112]. Therefore, additional clinical trials are necessary to investigate the anticancer effect of tea.

#### 4.4.4. Strategy to Improve Anticancer Effect of Tea and Its Component

Combinational therapies for cancer treatment have attracted increasing attention due to the inefficiency of single-drug treatment [113,114]. Synergistic effects may result in the enhanced anticancer activity of tea bioactive components. For instance, it was observed that oolong tea polyphenols and polysaccharides with high molecular weight had synergistic anticancer activity on hepatocellular carcinoma by inhibiting the proliferation and growth of cancer cells [113]. Dietary tea polyphenols also exerted a synergistic anticancer activity with bleomycin hydrochloride in human cervical cancer cells, through caspase-dependent and independent apoptotic pathways [114]. Moreover, the clinical success of using natural ingredients depends on efficient systemic delivery and bioavailability [115,116,117]. Singh et al. reported that poly (lactide-co-glycolide)-encapsulated tea polyphenols (theaflavin and EGCG) offered an up to 7-fold dose advantage regarding anti-proliferative activity in comparison to bulk theaflavin and EGCG, and also enhanced the apoptosis of cisplatin in different human cancer cells, like A549 lung carcinoma cells, HeLa cervical carcinoma cells, and THP-1 acute monocytic leukemia cells [115]. Mechanisms of action included the inhibition of NF-κB activation, induction of the cleavage of caspase-3/9 and Bax/Bcl2 ratio in favor of apoptosis, and inhibition of the expression of cyclin D1, MMP-9, and vascular endothelial growth factor (VEGF) that refers to cancer cell proliferation, metastasis, and angiogenesis, respectively. In addition, Mukherjee et al. reported that gold-conjugated green tea nanoparticles possessed more potent anticancer effect. The nanoparticles transformed the redox status, inhibited the Nrf2 (nuclear factor erythroid 2-related factor 2) activation, reduced the phosphorylation of IκB, blocked the nuclear translocation of NF-κB, and suppressed the NF-κB-dependent anti-apoptotic proteins Bcl2 and Akt, all of which triggered the onset of apoptosis in cancer cells [116].

**Figure 4:**
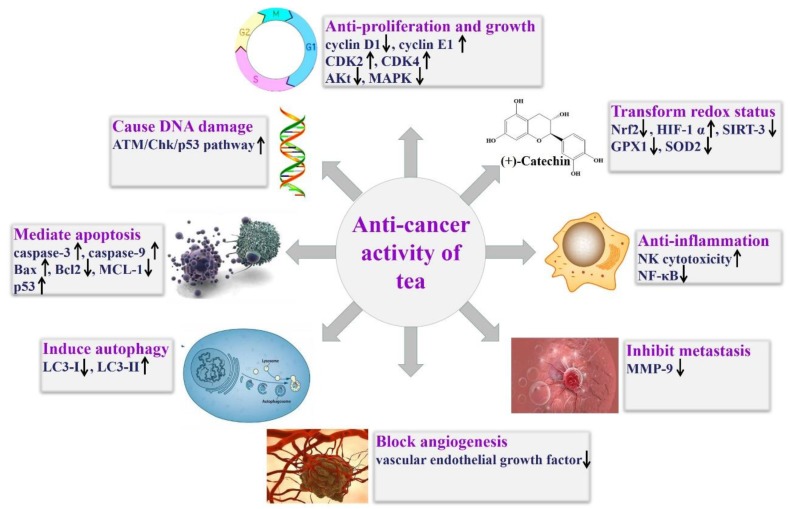
Main molecular targets of tea on targeting cancer. Abbreviations: Akt, protein kinase B; Bax, Bcl-2-associated X protein; Bcl-2, B-cell lymphoma 2; CAT, catalase; CDK, cyclin-dependent kinase; GPX, glutathione peroxidase; IL, interleukin; MAPK, mitogen-activated protein kinase; MCL-1, myeloid cell leukemia 1; MMP, matrix metallopeptidase; NF-κB, nuclear factor κB; NK, natural killer; SIRT, sirtuin; SOD, superoxide dismutase.

Overall, tea extracts and its components have shown protective effects against liver cancer, breast cancer, ovarian cancer, cervical cancer, prostate cancer, and leukemia, involving inhibiting initiation, proliferation, growth, resistance, metastasis, and angiogenesis, as well as inducing apoptosis, autophagy, and degradation of cancer cells, and related molecular targets are shown in Figure 4.

### 4.5. Cardiovascular-Protective Effect

Several epidemiological studies and meta-analyses suggest that tea brewing consumption is negatively correlated to the risk of CVD, such as hypertension, atherosclerosis, coronary heart disease, and angina [118,119]. Considering the Japanese Paradox, lower serum cholesterol level in the past Japanese middle-aged and elderly people compared to Western counterparts could help to maintain the low coronary heart disease incidence and mortality, in which reduced blood pressure level and smoking rate for both men and women also plays an important role. These can be helpful to explain the cardiovascular-protective effect of tea brewing and its components by targeting hyperlipidemia and hypertension [120].

#### 4.5.1. Cardiovascular-Protective Effect In Vitro

In an in vitro study, it was shown that Pu-erh tea (a kind of post-fermentation dark tea [7]) aqueous extract (PTAE) could regulate blood lipid metabolism enzymes, thereby ameliorating hyperlipidemia [121]. PTAE dose-dependently inhibited the activities of 3-hydroxy-3-methyl- glutaryl coenzyme A reductase (HMGR) and pancreatic lipase (PL) (PTAE acted as a competitive inhibitor) and lipoprotein-associated phospholipase A2 (Lp-PLA2) (PTAE acted as a non-competitive inhibitor), and increased the activity of lecithin:cholesterol acyltransferase (LCAT). In another study, Lung Chen Tea (a green tea) significantly inhibited endothelial cell-induced LDL oxidation as revealed by the reduced lipid peroxidation products, TBARS, and cellular cholesterol, thus may decrease the risk of coronary heart diseases [122]. Moreover, oolong tea extract was found to attenuate p-JNK (c-Jun N-terminal kinases) mediated hypertrophy, to suppress caspase-3-cleavage and apoptosis, to enhance the activities of IGF1R, Akt, and Bad, and to improve the Nrf2-mediated antioxidant system, all of which led to the prevention of cardiomyocyte loss against hypoxia [123].

#### 4.5.2. Cardiovascular-Protective Effect In Vivo

Tea and its components have been reported to lower blood pressure by some in vivo studies using animal models. Garcia et al. reported that intake of green tea (9.6 g in 1.0 L water; 18 mL/day) could reduce blood pressure, inhibit renal sympathetic nerve activity, improve arterial baroreceptor function, and ameliorate vascular and systemic oxidative stress in rats with hypertension induced by *N*-nitro-L-arginine-methyl-ester [124]. Moreover, the consumption of green tea extract (2 and 4 g/kg diet) was observed to benefit blood pressure and to improve inflammation and antioxidant status in NaCl-induced hypertensive rats [125]. However, results from another animal study argued that heavy tea consumption might be unsuitable for hypertensive subjects [126]. It was found that feeding of tea extract (300 mg/kg BW) induced an acute increase in systolic/diastolic blood pressure and heart rate in spontaneously hypertensive rats, which might be mediated by regulating plasma epinephrine and norepinephrine levels. Therefore, moderate tea consumption can be beneficial to hypertensive patients, but excessively heavy tea consumption might be harmful.

Some animal studies also demonstrated that tea and its extract also exerted protective effect against hyperlipidemia. For instance, aqueous extracts from fermented Pu-erh tea (150, 300, and 900 mg/kg BW) were shown to exhibit certain anti-hyperlipidemia effects in rats [127]. In addition, dried green tea leaves were mixed with 1% sucrose and 5 × 10^7^ colony-forming unit of *Bacillus subtilis* and fermented at 50 °C for 3 d, followed by further incubation at 90 °C for 4 d to remove remaining *B. subtilis* [128]. The results showed that the extracts (500 mg/kg BW) of fermentation of green tea exhibited a hypolipidemic effect by inhibiting PL, promoting energy expenditure, and reducing the proportion of the *Phylum Firmicutes* in the gut microbiota. Moreover, TPS (6.9 g/100 g diet) from green tea showed to suppress liver lipid accumulation and increase fecal excretion of dietary fat, which might help to reduce hyperlipidemia [129]. Meanwhile, TPS (100, 300, and 500 mg/kg BW) from Liupao tea (dark tea) dose-dependently increased antioxidant enzyme activities, ameliorated lipid oxidation, and improved the lipid profile in rats [130]. Besides, tea polyphenols (100 mg/kg BW) reduced lipid absorption by inhibiting lipase in the intestinal mucosa and contents, thus helped to prevent hyperlipidemia in rats treated with olive oil [131].

In the animal studies, tea consumption could ameliorate endothelial dysfunction and consequently benefit the vascular health [132,133,134]. Daily consumption of black tea (15 mg/kg BW, 4 weeks; active ingredients as theaflavins) exerted beneficial effects to reverse endothelial dysfunction in ovariectomized SD rats [132]. The mechanisms may involve improving flow-mediated dilatation in small mesenteric resistance arteries, augmenting acetylcholine (ACh)-induced endothelium-dependent relaxations in aortae and renal arteries, elevating ACh-stimulated cyclic guanosine monophosphate (cGMP) production in aortae, as well as restoring the phosphorylation of endothelial nitric oxide synthase (eNOS), the up-regulation of NADPH oxidases, and the overproduction of ROS in aortae. In addition, daily green tea EGCG treatment (50 mg/kg BW, 10 weeks) could improve endothelial function in high-fat diet-fed male C57BL/6J mice, by promoting insulin-stimulated vasodilation, restoring insulin-stimulated phosphorylation of eNOS, insulin receptor substrate-1, and Akt in primary bovine aortic endothelial cells, as well as reducing macrophage infiltration into aortic tissues [133].

#### 4.5.3. Cardiovascular-Protective Effect in Humans

Tea consumption can also reduce the risk of CVD in humans. It was reported that short-term daily consumption of three capsules containing 500 mg of green tea extract could reduce blood pressure in obese prehypertensive women [135]. In addition, tea consumption also improved the endothelial function in humans, and green and black teas may be equally effective with regard to improve endothelial function [136,137,138,139,140]. Acute black tea intake (200 mL/day) for seven days enhanced the cutaneous vascular response to gradual local heating to 42 °C in healthy, middle-aged participants, which was probably induced by activating endothelium-derived chemical mediators like NO [136]. The intake of black tea (with 150 mg polyphenols), twice a day for eight days, protected blood vessels in hypertensive patients through augmenting the amount of circulating angiogenic cells and blocking endothelial dysfunction [137]. Green tea catechins (580 mg/day, 2 weeks) improved human forearm endothelial dysfunction and exerted an antiatherosclerotic effect in smokers [138]. Green tea (equivalent to 200 mg EGCG/d) treatment was also observed to improve the endothelial function in humans in terms of flow-mediated dilation. However, its isolated EGCG might not contribute to this improvement [139]. Furthermore, tea consumption also reduced hyperlipidemia in humans [141,142]. Treatment with four green tea extracts capsules containing 1315 mg catechins (843 mg EGCG) daily for 12 months gave rise to a significant reduction of blood total cholesterol (TC) and LDL-cholesterol (LDL-C) levels, particularly in subjects with increased baseline TC level [141]. Similarly, daily consumption of functional black tea (with 2 g phytosterols) remarkably reduced the TC, LDL-C, and apolipoprotein B levels, as well as oxidative stress index in mild hypercholesterolemia subjects, while increased adiponectin and tissue-plasminogen activator and improved total antioxidant status [142]. However, in a diet-controlled randomized trial, daily intake of 5 cups of black tea had no significant alteration on the lipid profile of borderline hypercholesterolemic subjects [143].

Collectively, tea beverage consumption can decrease CVD risk, mainly by improving redox status, inhibiting inflammation, decreasing blood pressure, ameliorating hyperlipidemia, regulating endothelial function, preventing myocardial damage, and regulating sympathetic nerve activity. However, due to inconsistent results of human studies, more clinical trials that are rationally designed and accurately conducted are necessary to verify the cardiovascular-protective effect of tea.

### 4.6. Anti-Diabetic Effect

Postprandial hyperglycemia is one of the symptoms of type 2 diabetes mellitus (T2DM), and tea beverage consumption has been reported to ameliorate hyperglycemia, thus providing benefits to T2DM patients [144].

#### 4.6.1. Anti-Diabetic Effect In Vitro

Tea has been found to inhibit α-glucosidase, delay glucose absorption and reduce hyperglycemia in vitro [144,145,146]. Green, oolong, and black tea extracts exerted inhibitory activity against α-glucosidase, and green tea extracts exhibited the strongest effect [144]. Black tea aqueous extract significantly suppressed α-glucosidase activity and showed a mixed-type inhibitory activity with acarbose [145]. In addition, TPS was observed to inhibit α-glucosidase, which could be enhanced by removing the metal ions from the TPS [43,146]. Moreover, type II arabinogalactan, a water-soluble polysaccharide from green tea, could regulate the cyclic adenosine monophosphate-protein kinase A (cAMP-PKA) pathway and, correspondingly, significantly enhance glucose-stimulated insulin secretion in RIN-5F cells at a high glucose level (25 mM), but no effect was found at a low glucose level (5 mM) [47].

#### 4.6.2. Anti-Diabetic Effect In Vivo

Considering the health functions as shown in vivo, tea might serve as a functional food and pharmaceutical for the prevention and treatment of T2DM. Satoh et al. reported that black tea aqueous extract inhibited the hydrolysis of disaccharides into monosaccharides by the α-glucosidase in the small intestine, thereby blocking the sodium-dependent glucose cotransporter 1 and glucose transporter (GLUT) 2-mediated absorption of the dietary glucose [31]. Li et al. declared that TPS treatment could alleviate insulin resistance and decrease blood glucose in diabetic mice, which might be mediated by the regulation of the PI3K/Akt signal pathway as revealed by the up-regulated expression of PI3Kp85/p-Akt/GLUT4-signaling molecules [147]. Additionally, various kinds of tea might have different anti-hyperglycemic strength, and yellow tea might be a better choice compared to green and black teas [148]. In another study, mixtures of dried green tea leaves and *Aquilariae lignum* powder at weight ratio of 49:1 were wet-fermented for 12 h at 60 °C and steamed for 30 s at 100 °C after being dried for 1 week at 15 °C, then the steamed mixtures were cooled and additionally dried at 15 °C for 3 days [149]. The results showed that fermentation of green tea increased the anti-diabetic activity of green tea aqueous extracts in mouse fed with high fat diet, as revealed by the stronger hypoglycemic effect resulted from stronger inhibitory activity on the hepatic glucose-regulating enzymes including glucokinase, glucose-6-phosphatase, and phosphoenolpyruvate carboxykinase.

#### 4.6.3. Anti-Diabetic Effect in Humans

Tea has shown promising efficacy in managing T2DM in several clinical trials, in terms of improving insulin resistance and postprandial hyperglycemia of humans. Consumption of black tea significantly reduced glycated hemoglobin (HbA1c) level and helped to decrease the risk of suffering from TD2M in subjects [150]. In addition, regular intake of green tea could benefit high-fat diet-induced T2DM [151]. Meanwhile, green tea was found to augment the isomaltulose activity to reduce postprandial glucose and insulin concentration in healthy subjects [152]. Furthermore, supplementation with green tea extract could improve glycemic control and prevent osteoporosis in diabetic patients [153].

Thus, tea consumption can be a promising strategy for preventing and treating diabetes and its complications by regulating glucose absorption and metabolism, controlling postprandial glucose level, and ameliorating insulin resistance.

### 4.7. Anti-Obesity Effect

Recent studies have suggested an anti-obesity effect of tea and its components, partially by improving energy expenditure, lipid metabolism, and lipid accumulation.

#### 4.7.1. Anti-Obesity Effect In Vitro

Tea and its components could modulate the glycolipid digestion, absorption, and metabolism in vitro, resulting in a beneficial effect on obesity. White, green and black teas were found to effectively inhibit lipase activity, and dose-dependently reduce lipid deposition in cultured adipocytes [154]. In addition, a novel acylated flavonol tetraglycoside from Lu′an GuaPian tea (a kind of green tea) remarkably inhibited the proliferation, differentiation, and lipid accumulation of 3T3-l1 cells [33]. Total green tea polyphenols might exhibit a greater inhibitory effect than purified EGCG on adipogenesis in 3T3-l1 cells, through decreasing adipogenic factors, including CCAAT element-binding protein α, peroxisome proliferator-activated receptor γ (PPAR γ), and sterol regulatory element-binding protein-1c (SREBP-1c) [155].

#### 4.7.2. Anti-Obesity Effect In Vivo

Some results from in vivo studies have provided significant insights into the effects of tea for the prevention of obesity and related comorbidities like metabolic syndromes. Choi et al. reported that green tea extracts could ameliorate obesity, hepatic steatosis, dyslipidemia, and insulin resistance in diet-induced obese mice [156]. Supplementation of tea extract resulted in reduced body weight gain and adiposity through enhancing energy expenditure. The transcriptome profiles of epididymal white adipose tissue indicated that green tea augmented energy homeostasis by enhancing transcriptional reaction to the degradation of branched-chain amino acids, and by regulating adenosine monophosphate-activated protein kinase (AMPK) signaling. Moreover, green tea could increase the hepatic lysophosphatidylcholine acyltransferase 2/4 and, correspondingly, attenuate the reduction of several lipid metabolites in mice fed with a high-fat diet, such as lysophosphatidylcholine, lysophosphatidylethanolamine, and lysophosphatidylserine [157]. Furthermore, it was also demonstrated that polyphenols and polysaccharides were the major components contributing to the inhibitory effects of green tea extracts on body weight gain and fat accumulation in rats fed with a high-fat diet [48].

Regulation of the gut microbiota in obese animals can be a crucial component of the mechanism of the responses contributing to the anti-obesity effects of tea. Consumption of green, oolong and black teas markedly augmented the diversity and shifted the structure of the gut microbiota in high-fat-induced obese mice, including 30 key phylotypes, such as *Alistipes*, *Rikenella*, *Lachnospiraceae*, *Akkermansia*, *Bacteroides*, *Allobaculum,* and *Parabacteroides*, which could be closely correlated to the obesity-associated indexes [158]. Moreover, the administration of oolong tea polyphenols could protect against the obesity-related metabolic disorders by improving the expression of genes involved in the amino acid biosynthesis and carbon metabolism, and by manipulating the intestinal microbiota [159]. In detail, the increased abundance of butyrate- and acetate-producing bacteria, the large increase in *Bacteroidetes*, the decrease in *Firmicutes*, and the correspondingly decreased *Firmicutes*/*Bacteroidetes* ratio suggested the protective effect. Moreover, green tea polyphenols significantly blocked diet-induced weight gain, fat deposition, adipocyte hypertrophy, and hepatic steatosis in C57BL/6J human flora-associated mice, possibly by modulating the diversity of gut microbiota and by increasing the abundance of lactic acid bacteria [160].

#### 4.7.3. Anti-Obesity Effect in Humans

Tea has shown protective effects on obesity and related metabolic disorders in some clinical trials. It was reported that 12-week daily treatment with 856.8 mg green tea extracts gave rise to a large reduction in body weight, waist circumference, and plasma TC and LDL levels in central-obese women, without any detected adverse effect [161]. The mechanism might partially be due to inhibition of the secretion of ghrelin and raising the level of adiponectin. Taghizadeh et al. demonstrated that daily consumption of a mixture containing 125 mg green tea, 25 mg capsaicin, and 50 mg ginger extracts for eight weeks significantly reduced the weight, body mass index (BMI), plasma GSH level, and insulin metabolism markers in overweight women [162]. However, a 4-week decaffeinated green tea extract (571 mg/d) intervention did not alter total fatty acid concentrations in recreationally active males, although it enhanced substrate utilization and subsequent performance indices [163]. Furthermore, tea consumption in combination with exercise training might be a favorable strategy to control obesity. For instance, green tea supplementation (3 tablets of 500 mg after each main meal) with high-intensity interval training for 10 weeks could distinctly reduce body weight, BMI, and the undesirable consequence of overweight, through augmenting the levels of SIRT-1 and PPAR γ co-activator 1-α [164]. In addition, a 12-week intervention with three green tea capsules containing 250 mg of green tea extract (187.5 mg polyphenols, 125 mg EGCG, and 20 mg caffeine) daily and interval sprinting exercise significantly decreased body and abdominal fat, and increased total lean mass in overweight males [165].

In short, tea extract and its components, including polyphenols, caffeine and polysaccharides, showed potent anti-obesity effects, which could involve regulating glycolipid digestion, absorption, and metabolism, improving energy expenditure, preventing lipid accumulation and deposition, and ultimately reducing body weight gain and increasing lean mass.

### 4.8. Hepato-Protective Effect

Some natural products have been found to protect against liver injuries [166,167,168,169,170]. Tea has also shown a beneficial effect on diet- and chemical-induced disorders in liver, including hepatic oxidative stress damage, inflammation, steatosis, and fibrosis [41,171,172].

#### 4.8.1. Hepato-Protective Effect In Vitro

The increase of ROS and the depletion of the antioxidant defense system could evoke apoptosis in cultured hepatocytes, but the pretreatment with gold-conjugated green tea nanoparticles protected hepatocytes from cellular damage, with the mechanisms of scavenging excessive ROS, enhancing the activity of antioxidant enzymes, augmenting GSH level, as well as reducing Bax/Bcl2 ratio and active caspase-3 levels [116].

#### 4.8.2. Hepato-Protective Effect In Vivo

The hepato-protective effect of tea against chemical-induced liver injury was also demonstrated in several in vivo studies. White tea extract and the comparative dose of EGCG showed equivalent protective effects to attenuate benzo(a) pyrene-induced hepatic dysfunctions, in terms of increased biomarkers regarding inflammatory and oxidative stresses, decreased endogenous antioxidants, and the hepatic histoarchitectural alteration [172]. In addition, green tea could protect rats from alcohol-induced mitochondrial DNA damage, and could ameliorate oxidative stress by improving the activities of SOD, GPX, and CAT as well as increasing the content of GSH [173]. Moreover, theaflavin-enriched black tea extracts exerted a hepato-protective effect against dimethylnitrosamine-induced liver fibrosis in rats, probably via blocking the TGF-β1/Smad signaling [41].

Tea also possessed a hepato-protective effect against diet-induced liver injury, in particular the non-alcoholic fatty liver disease (NAFLD) [171,174]. Besides improving oxidative and inflammatory status, mechanisms also involved: (1) Increasing energy expenditure via enhancing mitochondrial complex chain; (2) inhibiting fat synthesis via modulating the mRNA expression of SREBP-1c, cAMP-response element-binding protein regulated transcription coactivator 2, and stearyl coenzyme A dehydrogenase-1; (3) improving cholesterol homeostasis via regulating the mRNA expression of apolipoprotein B100 and ATP-binding cassette transporter A1; and (4) preventing gut dysbacteriosis [18,175,176].

#### 4.8.3. Hepato-Protective Effect in Humans

Tea and its components have been considered as potential ingredients for ameliorating liver injuries in patients. In a double-blinded, placebo-controlled, randomized clinical trial, the green tea extract consumption (500 mg/day, 90 days) successfully decreased the levels of liver enzymes including ALT, AST, and ALP in patients with NAFLD [177]. In another clinical trial with hypercholesterolemic subjects, catechin-enriched green and oolong tea treatments were found to significantly decrease body weight, BMI, fat, lipid peroxidation, and lipid profiles (TG, TC, LDL-C, and HDL-C), while improving GSH, SOD, CAT, GPX, and GR in the liver [87].

Collectively, tea and its components show a hepato-protective effect, as they could ameliorate oxidative stress via improving the antioxidant defense system, inhibit inflammation, block liver cell apoptosis, regulate lipid metabolism, prevent hepatic steatosis and fibrosis, and retard gut dysbacteriosis.

### 4.9. Other Health Functions

Tea also exhibits some other health functions in vitro and *in vivo*, as shown in Table 2 and Table 3, respectively. For instance, tea could protect against kidney injuries induced by a high-fat diet, proline, gentamicin, lead, and ischemia-reperfusion [178,179,180,181,182,183]. In addition, tea has exhibited the neuro-protective effect, in terms of protecting against age-related neuro-degenerative disorders, depression and regulating the circadian clock [184,185,186,187,188,189,190]. Moreover, tea could inhibit gastric ulcer and improve gastrointestinal function [191,192,193,194,195]. Besides, tea could modulate gut microbiota composition (increase beneficial microorganisms and decrease harmful microorganisms), which might be beneficial to those in the risk of obesity, metabolic syndrome, hyperlipidemia, and cardiovascular diseases [196,197,198,199,200,201,202]. Furthermore, some studies have suggested the potent anti-bacterial, anti-fungal, and anti-viral activities of tea [34,56,61,203,204,205,206,207,208].

Taken together, tea brewing, extract and its bioactive components possess diverse health functions (Figure 1), such as antioxidant, anti-inflammatory, immuno-regulatory, anticancer, cardiovascular-protective, anti-diabetic, anti-obesity, and hepato-protective effects. Specifically, consumption of tea and its bioactive components has been reported with the potential to manage certain chronic diseases (Table 4), which could be helpful for establishing dietary guidelines for human beings to maintain good health, and for the utilization of tea as a raw material to develop functional beverages, nutraceuticals, and pharmaceuticals.

## 5. Potential Safety Issues

Food safety has attracted increasing attention due to its importance to human health. The safety issues of tea and its bioactive components should never be ignored despite its prominent health functions. There are several suspicions regarding the safety of tea, such as pollution by heavy metals, pesticide residues, and mycotoxin production during fermentation and storage, and the toxicity of high doses of its bioactive components [27,209,210,211] The potential safety issues of tea are summarized in Table 5.

Many elements have been detected in tea, including plumbum (Pb), cadmium (Cd), copper (Cu), cobalt (Co), chromium (Cr), nickel (Ni), manganese (Mn), aluminum (Al), arsenic (As), and fluorine (F) [212,214,225]. Some elements, such as Pb and Cd, are toxic, and others, such as Cu and F, are essential elements, but they can be harmful at high levels. Fortunately, the contents of these elements in tea were generally below the maximum permissible limits stipulated by the World Health Organization (WHO) and the United States Pharmacopeia (USP) [212]. In addition, both the target hazard quotient (THQ) and hazard index (HI) levels regarding these elements in certain teas were far below 1, indicating that tea consumption would not increase health risks [214,225]. Besides, the THQ and HI values would decrease as the infusion time increased, so it was suggested to discard the first tea infusion and consume the later ones [214]. In addition, some elements might be accumulated in tea during plantation, and the most strongly accumulated ones are Mn, Al, and F, which could induce poisoning symptoms, cognitive dysfunction, fluorosis of bone, and other adverse effects in humans [213,226,227]. Environmental and plant factors, such as the soil condition, variety, season, and maturity, might influence the contents and distribution of certain elements in tea. Thus, it is important to monitor these factors to assure that tea products do not contain excessive levels of heavy metals [226].

Excessive levels of pesticide residues, such as organophosphorus chemicals, organochlorines, carbamates, pyrethroids, herbicides, and neonicotinoids, are a serious safety issue that has been monitored for many decades and aroused increasing attention [216,219,228,229]. Specifically, chlorpyrifos, bifenthrin, lambda-cyhalothrin, cypermethrin, imidacloprid, and acetamiprid were frequently detected pesticides in tea [215,218]. Despite of the fact that the detection rates of pesticides in tea were relatively high, most of the investigated pesticides were below the Chinese and European Union maximum residue levels (MRLs) [25,215]. However, in one study, 39 out of 233 tested tea samples were found to exceed European Union MRLs, and bifenthrin had the highest detection rate [217]. On the other hand, the transfer rates of pesticides from tea leaves into the infusion should be taken into consideration when conducting risk assessments of pesticides in teas, since it could be affected by the solubility and polarity of pesticides, the temperature of the water, and brewing time [230,231,232]. In one study investigating 810 Chinese teas, the THQ values of organophosphorus pesticides from tea consumption were below 0.02, so the exposure of tea drinking-induced organophosphorus pesticide was unlikely to induce adverse health problems in humans [233].

Fermentation by a microbial consortium composed of bacteria, molds, and yeasts is probably the most important process in manufacturing some kinds of teas, in which a series of biochemical reactions occur, and the components are greatly transformed [7,234,235]. Some harmful fungi might bring potential safety hazards to the production of fermented teas during the fermentation stage as well as the storage period [220]. Aflatoxins, fumonisins, and ochratoxin A could be found in some tea samples, among which aflatoxin B1, the most toxic aflatoxin, could induce extremely serious hepatotoxicity, and it is not easy to remove in normal ways like flushing with water or heating at high temperature, even up to 200 ℃ [221]. Thus, it is important to monitor the fermentation process of tea so as to avoid the production of toxic metabolites.

Consumption of tea has been reported with few adverse events [127,148,236]. Tumorigenic, mutagenic, teratogenic, and sub-chronic toxicity of tea-related ingredients have not been observed in animal experiments and human studies, and the no-observed-adverse-effect level (NOAEL), observed safety level, and tolerable upper intake level were far from the functional doses [26,222,223,237]. However, some compounds contained in tea has been recognized as anti-nutritive factors. For example, tea tannins and polyphenols may inhibit digestive enzymes, including trypsin, lipase, amylase, and glucosidase that are related to digestion of protein, lipid, and carbohydrate, resulting in the decreased availability of these nutrients, which may do harm to malnutrition persons though benefit obesity or diabetic patients [238,239,240,241,242]. Excessive tea drinking may interfere with iron absorption and subsequently lead to iron deficiency anemia [243,244,245]. Anti-thiamine component in tea may destroy the bioactivity of thiamine, which cause neurological symptoms consisting of both peripheral and central nervous system dysfunction [246]. In addition, consumption of a high dose of tea extract/component (approximately up to 800 mg tea polyphenols) might cause mild acute adverse effects. High intake of caffeine (more than 200 mg/d) accompanied by tea consumption could cause, at least in sensitive peoples, short-term stimulation of the nervous system, insomnia, anxiety, palpitations, tremor, and increased blood pressure [247]. Some other harmful effect has also been reported to correlated with tea consumption, such as acute liver damage and gastrointestinal disturbances like nausea and stomach injuries, with the latter more likely to occur when the tea is consumed on an empty stomach instead of a full stomach [28,224]. Nevertheless, as demonstrated previously, consuming tea on an empty stomach can improve its bioavailability and consequently enhance its biological efficacy [77]. On the other hand, pretreatment with green tea EGCG may reduce the bioavailability as well as the hepato-toxic effect of subsequent oral bolus doses of EGCG [79]. Based on this discrepancy, considerable attention should be paid to keeping the balance between improving the bioavailability and bioactivity, as well as preventing the adverse effects of natural phytochemicals when investigating their health functions and safety and applying them as functional foods and pharmaceuticals in the future.

To sum up, tea is generally safe for consumption with very rare adverse effects. Rigorous regulations are essential to limit the toxic factors in tea products, including heavy metals, pesticide residues, and mycotoxins from plantation, manufacture, and storage.

## 6. Conclusions

In conclusion, polyphenols, polysaccharides, saponins, pigments, purine alkaloids, and free amino acids in tea can be the major bioactive components that contribute to its diverse health functions. Generally, the bioavailability of tea phytochemicals is relatively low, and some technologies, like modification technology, capsule technology, and nanotechnology, can improve their bioavailability, resulting in the increased bioactivities. The antioxidant, anti-inflammatory, immuno-regulatory, anticancer, cardiovascular-protective, anti-diabetic, anti-obesity, and hepato-protective effects have been widely demonstrated by in vitro, in vivo, and human studies, with various mechanisms of actions. Tea brewing and tea-based products are generally considered safe to consumers, as harmful impacts on animals and human have seldom been reported. Although heavy metals, pesticide residues, and mycotoxins could be detected in certain samples of tea, they are usually below the MRLs. Therefore, tea consumption can be recommended to the public for chronic disease prevention and treatment. In addition, tea can be processed into beverages, functional foods, and pharmaceuticals. In the future, the bioavailability of tea phytochemicals should be intensively investigated, and more technologies should be developed to improve their bioavailability. Underlying mechanisms of the health functions of tea, especially the molecular targets, need to be further clarified. In addition, more well-designed clinical trials are necessary to further verify its health functions. Besides, the synergetic effects of tea and its purified bioactive components with other food bioactive ingredients are worth further study to promote its health functions. Last but not least, more attention should also be paid to the safety of tea, such as contamination by heavy metals, pesticides, and mycotoxins, as well as the potential adverse impact of high doses of tea bioactive components. Overall, tea is a promising dietary component, and its consumption shows many health functions.

## Figures and Tables

**Table 1:** Phytochemical content (mg/g DW) of 6 representative teas from six categories [12].

Phytochemicals	Gongmei Tea	Dianqing Tea	Junshan Yinzhen Tea	Fenghuang Shuixian Tea	Yichang Congou Tea	Fuzhuan Brick Tea
	White Tea	Green Tea	Yellow Tea	Oolong Tea	Black Tea	Dark Tea
Catechin	ND	1.37	1.32	ND	ND	4.93
EC	ND	6.20	5.97	1.58	0.74	10.36
GC	ND	2.74	1.86	2.51	ND	5.54
EGC	8.42	13.66	13.09	31.25	ND	23.43
CG	ND	0.35	ND	ND	ND	ND
ECG	3.14	30.49	35.40	8.44	3.51	10.88
GCG	ND	1.45	ND	ND	0.51	0.93
EGCG	6.01	50.78	59.35	36.70	3.80	10.89
Galli acid	2.18	0.94	1.43	3.28	3.55	3.10
Chlorogenic acid	ND	ND	0.37	ND	0.19	0.28
Ellagic acid	ND	1.88	2.14	1.88	2.61	2.21
Kaempferol-3-G	0.50	1.05	1.61	1.19	1.45	1.00
Theaflavine	ND	ND	ND	ND	0.56	0.48
Caffeine	27.47	41.46	39.76	34.77	41.63	27.08

Notes: CG, catechin gallate; DW, dry weight; EC, epicatechin; ECG, epicatechin gallate; EGC, epigallocatechin; EGCG, epigallocatechin gallate; GC, gallocatechin; GCG, gallocatechin gallate; ND, not detected. Gongmei tea, Dianqing tea, Junshan Yinzhen tea, Fenghuang Shuixian tea, Yichang Congou tea, and Fuzhuan Brick tea are produced in Fujian, Yunnan, Hunan, Guangdong, Hubei, and Hubei provinces in China, respectively.

**Table 2:** Effects of tea and its components on microbes in vitro.

Ingredients	Dosages	Microbes	Effects	References
Polyphenols from green, oolong, and black teas	1% (*w*/*v*) in medium	Bacterium	Modulate intestinal flora, induce the proliferation of *Bifidobacterium* spp., and *Lactobacillus/Enterococcus* spp., and inhibit *Bacteroides-Prevotella* and *Clostridium histolyticum*.	[61]
Green tea	MIC: 400 μg/mL	Bacterium	Anti-bacterial activities against *Propionibacterium acnes, P. granulosum*, *Staphylococcus aureus,* and *S. epidermidis*	[206]
Green and black tea blend	MIC and MBC: 12.5 mg/mL	Bacterium	Anti-bacterial and bactericidal activities against *Streptococcus mutans.*	[203]
Green tea extract		Bacterium	Bactericidal activity against *Streptococcus mutans*.	[205]
23 tea extractions	MIC: 0.078–0.156 mg/mL	Fungus	Anti-fungal activities against *Candida glabrata, C. albicans* and *C. parapsilosis*.	[204]
Tea polyphenols, tea saponins and their combination	IC_50_: 1.66–2.92 mg/mL	Fungus	Inhibit the growth of *Rhizopus stolonifer* by inducing H_2_O_2_ production, leading to cell membrane oxidative damage and intracellular constituent leakage.	[56]
Tea gallic acid, GCG, Teavigo (>90% EGCG), and theaflavin-3,3′- digallate	15, 30, 60, 120 μmol/L gallic acid and 2.5, 5, 10, 20, 40 μmol/L GCG, Teavigo, and theaflavin- 3,3′-digallate	Fungus	Inhibit germination and outgrowth of *Bacillus subtilis* spores.	[207]
Green tea extract	0.5, 5 and 10 mg/mL	Virus	Inhibit enteric viruses including murine norovirus and hepatitis A virus.	[208]
Pu-erh tea ellagic acid	IC_50_: 6 μmol/L	Virus	Anti-viral activities against human influenza virus A/Puerto Rico/8/34.	[34]

Notes: EGCG, epigellocatechin gallate; GCG, gellocatechin gallate; MBC, minimum bactericidal concentration; MIC, minimum inhibitory concentration; IC_50_, 50% inhibitory concentration.

**Table 3:** Various in vivo effects of tea and its components.

Ingredients	Dosages	Subjects	Categories	Effects and Molecular Mechanisms	References
Green tea extract	1 mL/100 g BW, 1w	Rats	Reno-protection	Protect against proline-induced oxidative damage in the kidney.	[178]
Green tea extract	300 mg/kg BW, 15 d	Rats	Reno-protection	Ameliorate nephrotoxicity induced by gentamicin, by decreasing oxidative stress and lipid peroxidation in the kidney.	[180]
Polyphenols from green tea	20 or 50 mg/kg BW, 60 d	Wistar rats	Reno-protection	Protect against Pb-induced renal dysfunction and intoxication, by reducing Pb concentration and accumulation in kidney, suppressing apoptosis, scavenging ROS, inhibiting ROS-mediated ERK/JNK/p38 pathway and downstream cytokines.	[181]
Polyphenols from green tea	200 mg/kg BW, 18 d	Wistar rats	Reno-protection	Ameliorate high-fat diet-induced kidney injury, by regulating autophagy-lysosome related proteins (LC3-II, Beclin-1, p62, cathepsin B, cathepsin D, and LAMP-1) and elevating AMPK phosphorylation.	[182]
EGCG from green tea	50 mg/kg BW, once	SD rats	Reno-protection	Alleviate renal ischemia-reperfusion injury, by suppressing inflammation and cell apoptosis via regulating expression of TNF-α, IL-1 β, IL-6, Bax, and caspase-3.	[179]
EGCG from green tea	50 mg/kg BW, 3 w	129/svJ mice	Reno-protection	Ameliorate crescentic glomerulonephritis, by restoring Nrf2 activity and PPAR and SIRT1 levels, and decreasing p-Akt, p-JNK, p-ERK1/2, and p-P38.	[183]
Microbial metabolites of Chinese dark tea	10 mg/kg BW, 14 w	SAMP8 mice	Neuro-protection	Protect against age-related neurodegenerative disorders, by down-regulating the formation of 4-HNE and ubiquitinated protein aggregates and the Aβ metabolic pathway, increasing endogenous anti-oxidant capacity, relieving cell hypoxia, and reducing the rate of neuronal apoptosis.	[185]
Black tea	1.5% in drinking water, 60 d	Wistar rats	Neuro-protection	Protect against AD induced by AlCl_3_, attenuated cognitive deficits, by improving beta-amyloid 1–42, acetylcholinesterase, TBARS, GSH, SOD, CAT, GPX, Bax, Bc1-2, cyto c, and caspases-3/8/9 in hippocampus and cortex.	[189]
Green tea	1333 mg/mL in drinking water, 8 w	Rats	Neuro-protection	Protect against AD, avoided memory deficits, by preventing oxidative stress and damage in the hippocampus.	[187]
Green tea	2 g/2 pills/d, 2 m	Patients with AD	Neuro-protection	The benefit to cognitive function, by enhancing anti-oxidant system.	[184]
Polyphenols form green tea	2 g/L in drinking water, 8 w	C57BL/6J mice	Neuro-protection	Ameliorate memory impairment, by reversing the relatively shallow daily oscillations of circadian clock genes transcription and protein expression in both liver and hypothalamus.	[190]
GABA from green tea	0.83, 1.67, or 3.33 g/kg BW, 15 d	Mice	Neuro-protection	Reduce depression, by modulating GABAergic neurotransmission of cerebral cortex via up-regulating the expression of GABA(A) receptor α 1.	[188]
GABA green tea	50 and 100 mg/kg BW, 7 d	Balb/c mice	Neuro-protection	Reduce depression in post-stroke depressive mice, by reducing oxidative stress via improving endogenous anti-oxidant system.	[186]
Pu-erh tea	0.50, 1.00, or 1.50 g/kg BW, 14 d	SD rats	Gastrointestinal-protection	Ameliorate gastric ulcer, by decreasing the activity of myeloperoxidase and the concentration of asymmetric arginine in gastric mucosal homogenate.	[194]
Hetero-polysaccharides from green and black teas		Wistar rats	Gastrointestinal-protection	Ameliorate gastric ulcer, by protecting gastric mucosa, reducing gastric lesions, and maintaining gastric mucus.	[192]
Polyphenols from dragon pearl tea	50, 100, or 200 mg/kg BW, 4 w	Mice	Gastrointestinal-protection	Ameliorate gastric ulcer, by improving stomach acidity conditions, altering serum levels of SOD, GPX, CAT, MDA, and lipid peroxidation, increasing the mRNA expression levels of epidermal growth factor, epidermal growth factor receptor, vascular endothelial growth factor, and vascular endothelial growth factor receptor 1, and reducing gastrin expression levels.	[195]
Fuzhuan brick-tea	200 mg/kg BW, 8 w	Rats	Gastrointestinal-protection	Improve the intestinal function of high-fat diet-fed to rats, by increasing two *Lactobacillus spp* in intestinal microbiota.	[191]
Fuzhuan brick-tea	1, 10, or 20 g/kg BW, 10 d	Kunming mice	Gastrointestinal-protection	Regulate colonic microbiota, increased species diversity in *Lactobacillus*, *Bacteroides*, and *Clostridium* cluster IV.	[193]
Ripped Pu-erh tea extract	0.1%, 0.2%, or 0.4% in tap water, 8 w	Male C57BL/6N mice		Decrease weight gain, fat accumulation, adipose inflammation, and metabolic endotoxemia while improving the intestinal barrier integrity, by modulating gut microbiota composition (decreasing the *Firmicutes*/*Bacteroidetes* ratio).	[196]
Water extracts of green, oolong, and black teas	1% in drinking water, 28 w	C57BL/6J mice	Gut microbiota modulation	Reduce gain in weight, hepatic lipid, and white adipose tissue weight and plasma level of LPS, increase production of short-chain fatty acids, by regulating gut microbiota composition (decreasing the relative abundance of family *Rikenellaceae* and *Desulfovibrionaceae* and changing the abundance of key operational taxonomic units including *Alistipes*, *Rikenella*, *Ruminiclostridium*, and *Acetatifactor*).	[197]
Polyphenols from green tea	0.1% in diet, 8 w	C57BL/6J mice	Gut microbiota modulation	Ameliorate the obesity-induced gut dysbiosis, decrease the *Firmicutes/Bacteroidetes* ratio.	[198]
Extract of Fuzhuan brick-tea	400 mg/kg BW, 8 w	C57BL/6J mice	Gut microbiota modulation	Improve oxidative injury, inflammation, lipid metabolism, and obesity, by enhancing the diversity of gut microbiota, reducing the *Firmicutes*/*Bacteroidetes* ratio, and enhancing the relative abundance of *Bifidobacteriaceae*.	[199]
Polysaccharides from Fuzhuan brick tea	200, 400, or 800 mg/kg BW, 8 w	C57BL/6 mice	Gut microbiota modulation	Increase phylogenetic diversity of gut microbiota, restore the HFD-induced increases in relative abundances of *Erysipelotrichaceae*, *Coriobacteriaceae*, and *Streptococcaceae*.	[201]
Polyphenols from green tea	0.5% and 1.5% in drinking water, 3 or 6 m	SD rats	Gut microbiota modulation	Modify gut-microbiota dependent metabolisms of energy, bile constituents, and micronutrients	[200]
Tea polyphenols	100, 200, or 400 mg/kg BW, 12 w	C57BL/6 mice	Gut microbiota modulation	Ameliorate hyperlipidemia, improve the expression levels of hepatic lipid metabolism genes, and modulate gut microbiota, by modulating intestinal redox state.	[202]

Notes: 4-HNE, 4-Hydroxynonenal; AD, Alzheimer′s disease; Akt, protein kinase B; AMPK, adenosine monophosphate-activated protein kinase; Bax, Bcl-2-associated X protein; Bc1-2, B-cell lymphoma 2; CAT, catalase; EGCG, epigallocatechin gallate; ERK, extracellular signal–regulated kinases; GABA, gamma-aminobutyric acid; GPX, glutathione peroxidase; GSH, reduced glutathione; HFD, high fat diet; IL, interleukins; JNK, c-Jun N-terminal kinases; LAMP-1, lysosomal-associated membrane protein 1; LC3-II, light chain 3-II; LPS, lipopolysaccharide; MDA, malonaldehyde; Nrf2, nuclear factor erythroid 2-related factor 2; PPAR, peroxisome proliferator-activated receptor; ROS, reactive oxygen species; SOD, superoxide dismutase; SIRT1, sirtuin 1; TBARS, thiobarbituric acid reactive substances; TNF-α, tumor necrosis factor α.

**Table 4:** Health functions of tea in clinical trials.

Subjects	Ingredients	Dosages	Health Functions	Mechanisms	References
60 male sprinters	Green tea extract	2 capsules × times/d, 2 × 4 w, with a 4-week washout period	Antioxidation	Prevent oxidative stress, by increasing total antioxidant capacity and decreasing MDA level of blood plasma.	[85]
60 mildly hyper-cholesterolemic subjects	Catechin- enriched green and oolong tea	2 × 300 mL/d, 12 w	Antioxidation	Improve GSH, SOD, CAT, GPx, and GR, and decrease lipid peroxidation.	[87]
32 participants	Benifuuki and Yabukita green tea	3 cups/d, 2 w	Antioxidation	Protect against cutaneous oxidative stress, by increasing the radical scavenging activity of the skin.	[86]
68 SLE patients	Green tea extract	1000 mg/2 capsules/d, 12 w	Anti-inflammation	Improve the SLE disease as well as the corresponding vitality and general health.	[94]
45 male soldiers	Green tea	12 g tea leaves/d	Anti-inflammation	Decrease plasma levels of IL-6 and NF-κB in soldiers with sleep deprivation.	[95]
9 well-trained male cyclists	Green tea and carbohydrate	Acute ingestion	Anti-inflammation	Did not evidently improve inflammatory biomarkers during sprint cycling in athletes in comparison to carbohydrates.	[96]
16 tobacco smokers	Green tea	5 × 1 cup/d, 4 w	Anticancer	Reduce the risk of oral carcinogenesis, by modulating oral bacteria.	[110]
70 Algerian prostate cancer patients and 120 age-matched healthy subjects	Green tea	5 cups/2 g tea leaves/d, 6 m	Anticancer	Prevent prostate cancer initiation or delay its progression.	[111]
60 high-grade prostate intraepithelial neoplasia patients	Green tea catechins	600 mg/d, 6 and 12 m	Anticancer	Show a non-significant improvement in lower urinary tract symptoms and a better quality of life with very limited adverse effects.	[112]
20 obese prehypertensive women	Green tea extract	500 mg/3 capsules/d, 4 w	Cardiovascular-protection	Reduce blood pressure.	[135]
20 healthy participants	Black tea	200 mL/d, 1 w	Cardiovascular-protection	Enhance the cutaneous vascular response to gradual local heating to 42 °C, by activating endothelium-derived chemical mediators like NO.	[136]
19 hypertensive patients	Black tea	With 150 mg polyphenols, twice/d, 8 d	Cardiovascular-protection	Protect blood vessels, by augmenting the amount of circulating angiogenic cells and blocking endothelial dysfunction.	[137]
30 healthy male smokers	Green tea catechins	580 mg/d, 2 w	Cardiovascular-protection	Improve human forearm endothelial dysfunction, and anti-atherosclerosis.	[138]
50 healthy men	Green tea	equivalent to 200 mg EGCG/d	Cardiovascular-protection	Improve the endothelial function in humans in terms of flow-mediated dilation.	[139]
936 postmenopausal women	Green tea extracts	1315 mg catechins/4 capsules/d, 12 m	Cardiovascular-protection	Reduce blood TC and LDL-C levels, particularly in subjects with increased baseline TC level.	[141]
99 mild hyper-cholesterolemia subjects	Functional black tea	with 2 g phytosterols, once/d, 4 w	Cardiovascular-protection	Reduce the TC, LDL-C, and apolipoprotein B levels, as well as oxidative stress index, increase adiponectin and tissue-plasminogen activator, and improve total antioxidant status.	[142]
57 borderline hypercholesterolemic individuals	Black tea	5 cups/d, 4 w	Cardiovascular-protection	Show no significant alteration on the lipid profile.	[143]
30 T2DM patients	Black tea	1 or 3 cups (200 or 600 mL)/d, 12 w	Anti-diabetes	Reduce HbA1c level and help to decrease the risk of suffering from TD2M.	[150]
15 healthy subjects	Green tea	400 mL/visit, 5 visits with a two-week washout period	Anti-diabetes	Suppress postprandial plasma glucose and insulin concentration.	[152]
35 diabetic subjects	Green tea extract	1120 mg/d, 10 and 20 w	Anti-diabetes	Improve glycemic control and prevent osteoporosis in diabetic patients	[153]
102 women with central obesity	Green tea extracts	856.8 mg/d, 12 w	Anti-obesity	Reduce body weight, waist circumference, and plasma TC and LDL levels, probably by inhibiting ghrelin secretion and increasing adiponectin levels.	[161]
50 overweight women	Mixture of extracts	125 mg green tea, 25 mg capsaicin, and 50 mg ginger extracts/d, 8 w	Anti-obesity	Reduce the weight, BMI, plasma GSH level, and insulin metabolism markers.	[162]
30 non-athlete overweight females	green tea	500 mg/3 tablets/d with high- intensity interval training, 10 w	Anti-obesity	Reduce body weight, BMI, and the undesirable consequence of overweight, by augmenting the levels of SIRT-1 and PPAR γ co-activator 1-α.	[164]
48 overweight males	Green tea extracts	250 mg/3 capsules/d, with interval sprinting exercise, 12 w	Anti-obesity	Decrease body and abdominal fat, and increase total lean mass in overweight males	[165]
80 participants with NAFLD	Green tea extract	500 mg/d, 90 d	Hepato- protection	Decrease the levels of liver enzymes including ALT, AST, and ALP	[177]
60 mildly hypercholesterolemic subjects	Catechin- enriched green and oolong teas	2 × 300 mL/d, 12 w	Hepato- protection	Decrease body weight, BMI, fat, lipid peroxidation, and lipid profiles (TG, TC, LDL-C, and HDL-C), and improve GSH, SOD, CAT, GPX, and GR in the liver.	[87]

Notes: ALP, alkaline phosphatase; ALT, alanine aminotransferase; AST, aspartate transaminase; BMI, body mass index; CAT, catalase; GPx, glutathione peroxidase; GR, glutathione reductase; GSH, reduced glutathione; HbA1c, glycated hemoglobin A1C; HDL-C, high-density lipoprotein-cholesterol; IL, interleukins; LDL-C, low-density lipoprotein-cholesterol; MDA, malonaldehyde; NF-κB, nuclear factor-κB; PPAR γ, peroxisome proliferator-activated receptor γ; SIRT1, sirtuin 1; SLE, systemic lupus erythematosus; SOD, superoxide dismutase; T2DM, type II diabetes mellitus; TC, total cholesterol; TG, triglyceride.

**Table 5:** Potential safety issues of tea.

Samples	Location	Safety Categories	Specific Safety Items	Remarks	References
15 teas	Ghana	Heavy metal	Fe, Cu, Zn, Pb, As, and Cd	Below the maximum permissible limits by WHO and USP.	[212]
26 teas	Guizhou, China		Pb, Cu, As, Hg, Cd, and Cr	Below the standard limit values in China.	[213]
26 green teas	Jiangxi, China		Cd, Cr, Pb, and Cu	Cu content (31.48 mg/kg) in one sample exceeded the maximum allowable levels (30 mg/kg) for tea.	[214]
100 Pu-erh teas	Yunnan, China	Pesticide	74 pesticides	11 pesticides were detected, below the Chinese maximum residual levels.	[215]
6 teas	Different regions of China		Lindane, Parathion-Methyl, Methidathion, Fenitrothion, Fenthion, Fenpropathrin, Endosulfan sulfate, α-Endosulfan, β-Endosulfan, P,P’-DDE, O,P’-DDT, P,P’-DDD, P,P’-DDT, Bifenthrin, Permethrin	Below the MRLs by European Union.	[216]
223 teas	Yunnan, Zhejiang, and Fujian, China		32 pesticides	Residue levels in 39 samples exceeded the MRLs by European Union.	[217]
24 teas	Beijing, China		15 classes of pesticides	Chlorpyrifos (145.1 μg/kg) and α-HCH (22.2 μg/kg) in green tea exceeded the European Union MRLs (100 and 20 μg/kg, respectively).	[218]
8 teas	Beijing, China		Methomyl, Dimethoate, Propoxur, Carbaryl, Pirimicarb, Malathion, Fenitrothion, Kresoxim-methyl, Bifenthrin, Chlorpyrifos, Fenpropathrin, Lambda-cyhalothrin, Cypermethrin, Deltamethrin, Fenvalerate, Carbosulfan	Methomyl (197.45 ng/g *vs*. 100 ng/g) in black tea exceeded the MRLs by European Union.	[219]
18 teas	Almería, Spain	Mycotoxin	Aflatoxins	The aflatoxin B1 (5.4 mu g/kg) was found in one of the green tea samples.	[220]
36 Pu-erh teas	Yunnan, China		Aflatoxins, fumonisins, and ochratoxins	Ochratoxin A was detected in 4 of 36 teas (11.1%).	[221]
Green tea extract		Toxicity	Mutagenic and genotoxic toxicity	No targeted effects were observed.	[222]
Black tea extract			Acute and sub-chronic toxicity	No targeted effects were observed.	[223]
Green tea extract			Gastrointestinal symptom	6.7% of the participants experienced alanine aminotransferase elevations, with 1.3% experiencing alanine aminotransferase-related serious adverse events.	[224]

Notes: DDD, dichlorodiphenyldichloroethane; DDE, dichlorodiphenyldichloroethylene; DDT, dichlorodiphenyltrichloroethane; HCH, hexachlorocyclohexane; MRLs, maximum residue levels; USP, United States Pharmacopeia; WHO, world health organization.
